# Wastewater Respiratory Virus Surveillance in Remote Community, Alaska, USA, 2022–2024

**DOI:** 10.3201/eid3213.260709

**Published:** 2026-08

**Authors:** Brian Lefferts, Alyssa Leary, Dana Bruden, Ian Blake, Christine Richman, Sarah Sixberry, Michael Vicente, Jennifer Pak, Ellen Hodges, James W. Keck

**Affiliations:** Yukon-Kuskokwim Health Corporation, Bethel, Alaska, USA (B. Lefferts, A. Leary, C. Richman, S. Sixberry, M. Vicente, J. Pak, E. Hodges); Centers for Disease Control and Prevention, Anchorage, Alaska, USA (D. Bruden, I. Blake, J.W. Keck); Alaska Native Tribal Health Consortium, Anchorage (J.W. Keck)

**Keywords:** Viruses, respiratory infections, influenza, wastewater, surveillance, respiratory syncytial virus, SARS-CoV-2, American Indian or Alaska Native, rural population, rural health services, Alaska, United States

## Abstract

Wastewater surveillance (WS) is underused in rural communities. We evaluated WS performance in the remote, subarctic, mostly Indigenous, community of Bethel, Alaska, USA, during October 2022–May 2024. Wastewater was collected >3 times weekly and underwent on-site PCR testing for SARS-CoV-2, respiratory syncytial virus (RSV), and influenza A and B viruses. We compared WS virus detection with local clinical data by using results from 318 wastewater samples and 7,392 clinical tests. We detected SARS-CoV-2 in 265 (83.3%) wastewater samples, influenza A virus in 128 (40.3%), RSV in 78 (24.5%), and influenza B virus in 29 (9.1%). Wastewater signals correlated with clinical results for influenza B virus (Spearman ρ = 0.85), influenza A virus (ρ = 0.62), RSV (ρ = 0.60), and SARS-CoV-2 (ρ = 0.59), providing corroborating data that informed the timing of seasonal respiratory virus immunization campaigns. Our findings show that WS is feasible and useful for disease surveillance in rural Alaska.

Wastewater surveillance (WS) expanded substantially during the COVID-19 pandemic (*1*). Testing wastewater for the SARS-CoV-2 virus has several advantages over traditional case-based disease surveillance, including its efficiency in monitoring community disease trends, independence from healthcare-seeking behaviors that inform traditional surveillance approaches, and scalability from building-level monitoring to sewersheds serving millions of persons (*2*). WS has yielded actionable COVID-19 public health information across a variety of settings (*3*). Subsequently, WS platforms have expanded to include additional respiratory viruses, such as respiratory syncytial virus (RSV) and influenza virus (*4*), and have supported public health responses during respiratory virus seasons (*5*).

Smaller and remote communities have had lower uptake of WS (*6*). Barriers to adoption in rural and remote areas include limited access to wastewater testing laboratories, costs, insufficient human resources, lack of centralized wastewater collection and treatment systems, and competing priorities (*7*). Local implementation of WS could overcome the high cost and logistical challenges of shipping samples to third-party laboratories, thus improving the timeliness of wastewater data (*8*). Early in the COVID-19 pandemic, a team from Canada demonstrated the feasibility and accuracy of using a rapid and user-friendly clinical PCR platform to measure SARS-CoV-2 virus levels on site in a remote community (*9*). Their WS pilot uncovered previously unknown COVID-19 infections in the community and initiated a public health response. The reported effectiveness and relative simplicity of the WS approach used by Canada team informed the implementation of WS in a remote community in Alaska, USA.

Rural Alaska is home to hundreds of mostly Indigenous communities located off the road system, relying on water and air transportation for travel. Historic inequities and socio-environmental conditions, such as lack of in-home piped water and household crowding, have contributed to high rates of respiratory virus infections, particularly in rural southwest Alaska (*10*). The Yukon-Kuskokwim Health Corporation (YKHC) is a comprehensive regional Tribal Health Organization that provides most of the healthcare in the region. YKHC responded proactively to the COVID-19 pandemic with timely vaccine campaigns, physical distancing measures, and an expansive clinical testing and contact tracing program (*11*,*12*). As pandemic response measures transitioned to routine public health activities, YKHC invested in WS to monitor respiratory virus trends in the community. We analyzed 2 years of WS data for SARS-CoV-2, RSV, and influenza A and B viruses and compared wastewater viral signals with clinical testing data from the YKHC-operated YK Delta Regional Hospital.

## Methods

### Study Setting

YKHC initiated WS on October 19, 2022, in the regional hub community of Bethel, Alaska. Bethel is located 400 miles west of Anchorage in southwestern Alaska and is home to ≈6,300 residents living in 2,450 households; 74% of residents identify as Alaska Native. Many of the additional 22,000 residents in the region regularly pass through Bethel because it serves as the transportation hub and has the only hospital in the region.

Wastewater management in Bethel is complex and is served by 2 different systems: a piped sewer system in the central part of town and a truck-hauled system for households and businesses outside that area. The piped system accounts for 25% of residential and commercial connections and serves 479 customers, including the YK Delta Regional Hospital. Piped services account for ≈65% of the wastewater produced by volume in the community. In contrast, the 1,373 residential and commercial hauled customers account for 75% of connections but produce only 35% of Bethel’s wastewater. Hauled customers rely on 3,000-gallon trucks to deliver water to onsite storage tanks; wastewater trucks haul away wastewater to dump into the centralized sewer system or the sewage lagoon. Hauled services are expensive to operate, resulting in higher utility costs, which causes hauled customers to minimize water use.

We obtained wastewater samples from Bethel’s last lift station, which pumps raw sewage to a higher elevation so it can flow via gravity into the Bethel sewage lagoon. Previous research identified stronger viral signals from lift station samples than lagoon samples (*13*). Approximately 90% of piped wastewater in Bethel passes through that lift station, including sewage from the regional hospital and long-term care facilities. The only piped wastewater that does not pass through that site is from the Lower Kuskokwim School District campus, which discharges its sewage into the lagoon. Approximately 50% of Bethel’s hauled sewage also passes through that lift station.

### Wastewater Sampling

During October 2022–May 2024, we passively sampled wastewater by using the Moore swab method (*14*) (Appendix). During October 19, 2022–January 24, 2023, and June 28–September 19, 2023, sampling frequency ranged from 1 to 3 (mean 1.7) times/week. During January 25–June 27, 2023, and September 20, 2023–May 31, 2024, sampling frequency ranged from 3 to 5 (mean 4.7) times/week. Initially, we conducted sampling once per week while we developed collection and processing methods; we also reduced sampling to 1 time/week during periods of low respiratory virus activity. Sampling frequency increased with virus activity but varied depending on staffing and supply constraints. We collected Moore swabs 24 hours after placement except for swabs placed on Fridays, which the team collected the following Monday, resulting in 72-hour samples.

### Wastewater Analysis

We analyzed wastewater samples via real-time PCR on a GeneXpert Xpress with Xpert Xpress CoV-2/Flu/RSV plus viral respiratory cartridges (both Cepheid, https://www.cepheid.com), which test for SARS-CoV-2, influenza A and B viruses, and RSV. That system includes sample processing and probe check controls and reports results as cycle threshold (Ct) values. Scientists with the Public Health Agency of Canada validated the use of a clinical testing platform for wastewater analysis (*9*), but YKHC did not validate this laboratory method.

We evaluated a concentration step before PCR analysis in samples collected during October 2022–May 31, 2023 (Appendix). However, we elected to use unconcentrated samples for surveillance because the concentration step required additional resources and time without notable improvement in virus detection (Appendix Tables 1, 2). To analyze the unconcentrated wastewater samples, we added 300 µL of wastewater to a Cepheid GeneXpert cartridge and followed the manufacturer’s protocol for testing clinical specimens.

### Clinical Testing

Clinical respiratory virus testing followed YKHC standards of care. Patients of all ages with respiratory symptoms in the ambulatory, emergency, or inpatient settings at the YK Delta Regional Hospital (the only hospital in the region) were tested via collection of midturbinate nasal swab sample as part of routine clinical care. Swab samples were analyzed in the hospital’s medical laboratory by using the Cepheid GeneXpert respiratory panel kit to detect SARS-CoV-2, influenza A and B viruses, and RSV. We extracted deidentified clinical testing data from YKHC’s electronic health record system. During the study, some community members reported results from SARS-CoV-2 tests to the YKHC Department of Public Health. We conducted a separate correlational analysis by using self-reported SARS-CoV-2 test results. We considered positive virus testing results or self-reported results occurring within 30 days of a prior confirmed positive result for the same virus part of the same illness and excluded those results from the analysis.

### Statistical Analyses

We performed analyses for the entire evaluation period, partitioning our data into 2 respiratory virus seasons because of year-to-year variability in respiratory virus circulation. We followed respiratory virus activity levels from the Centers for Disease Control and Prevention to define respiratory virus season as week 27 to week 26 in the following year (*15*) and adjusted for seasonal onset and offset of WS at our site. Hence, the 2022–23 season included wastewater samples collected during October 19, 2022–June 30, 2023, and the 2023–24 season included samples collected during July 1, 2023–May 31, 2024. We calculated 7-day moving averages for virus Ct values from wastewater samples and for clinical test results. For wastewater samples spanning multiple days, we averaged Ct values for each day sampled. Spearman rank-order correlation (ρ) assessed the association between positive clinical test counts and wastewater Ct values. We conducted time-lag correlation analyses for each respiratory virus overall and by season using 7-day moving averages from wastewater and clinical data. We shifted wastewater Ct values from 10 days before (i.e., preceding) to 4 days after (i.e., lagging) the clinical testing data series. Of note, a lower Ct value indicates a higher relative viral concentration in the wastewater sample, which when correlated with the frequency of positive clinical tests yields negative correlation coefficients. We report absolute values of those correlation coefficients in the results for ease of interpretation; a larger coefficient represents a stronger correlation.

We evaluated the sensitivity, specificity, and concordance of the detection of each virus in wastewater with reference to its detection in clinical testing data. We separately aggregated wastewater and clinical data by surveillance week and virus, comparing presence (positive test) and absence (no positive test). We used contingency tables to organize the data with clinical testing serving as the reference standard. Because the GeneXpert platform runs 45 cycles, we defined a Ct value <45 as a positive wastewater test and used unlagged data. Influenza B analysis was limited to the 2023–24 season because influenza B was not detected in clinical or wastewater samples during the 2022–23 surveillance season.

This evaluation was deemed research not involving human subjects (45 CFR 46.102(e)) by the Centers for Disease Control and Prevention. YKHC and the Alaska Native Tribal Health Consortium approved this project.

## Results

### Wastewater and Clinical Virus Tests

YKHC initiated wastewater sampling 321 times during October 19, 2022–May 31, 2024, and PCR viral test results from 318 samples (99.1%) were available; in 3 instances, the Moore swab was lost in the wastewater stream during the passive sampling process. During the same timeframe, a total of 7,392 clinical respiratory tests detected influenza A virus 543 times (7.3% positivity), SARS-CoV-2 372 times (5.0% positivity), RSV 364 times (4.9% positivity), and influenza B 107 times (1.4% positivity) (Table). YKHC received 779 self-reports of positive SARS-CoV-2 tests.

**Table:** Results of wastewater and clinical respiratory virus testing for surveillance in remote community, Bethel, Alaska, USA, 2022–2024*

Season†	RSV	Influenza A virus	Influenza B virus	SARS-CoV-2
Overall surveillance period				
Samples				
Wastewater, n = 318	78 (24.5)	128 (40.3)	29 (9.1)	265 (83.3)
Clinical, n = 7,392	364 (4.9)	543 (7.3)	107 (1.4)	372 (5.0)
Surveillance days				
Wastewater, n = 503	111 (22.1)	185 (36.8)	41 (8.2)	406 (80.7)
Clinical, n = 581	215 (37.0)	203 (34.9)	49 (8.4)	232 (39.9)
Surveillance weeks, n = 85				
Wastewater	34 (40.0)	52 (61.2)	9 (10.6)	78 (91.8)
Clinical	60 (70.6)	57 (67.1)	12 (14.1)	73 (85.9)
2022–23 season				
Samples				
Wastewater, n = 135	18 (13.3)	33 (24.4)	0	129 (95.6)
Clinical, n = 3,781	228 (6.0)	303 (8.0)	0	193 (5.1)
Surveillance days				
Wastewater, n = 210	29 (13.8)	55 (26.2)	0	192 (91.4)
Clinical, n = 255	119 (46.7)	79 (31.0)	0	126 (49.4)
Surveillance weeks, n = 37				
Wastewater	11 (29.7)	19 (51.4)	0	37 (100)
Clinical	30 (81.1)	21 (56.8)	0	36 (97.3)
First detection date				
Wastewater	2022 Nov 7	2022 Nov 7	NA	2022 Oct 19‡
Clinical	2022 Oct 29	2022 Oct 21	NA	2022 Oct 20
Last detection date				
Wastewater	2023 Apr 26	2023 Jun 14	NA	2023 Jun 26
Clinical	2023 Jun 12	2023 Jun 3	NA	2023 Jun 20
2023–24 season				
Samples				
Wastewater, n = 183	60 (32.8)	95 (51.9)	29 (15.8)	136 (74.3)
Clinical, n = 3,611	136 (3.8)	240 (6.6)	107 (3.0)	179 (5.0)
Surveillance days				
Wastewater, n = 293	82 (28.0)	130 (44.4)	41 (14.0)	214 (73.0)
Clinical, n = 326	96 (29.4)	124 (38.0)	49 (15.0)	106 (32.5)
Surveillance weeks, n = 48				
Wastewater	23 (47.9)	33 (68.8)	9 (18.8)	41 (85.4)
Clinical	30 (62.5)	36 (75.0)	12 (25.0)	37 (77.1)
First detection date				
Wastewater	2023 Sep 26	2023 Jul 3	2024 Mar 21	2023 Jul 3
Clinical	2023 Oct 9	2023 Jul 23	2024 Jan 5	2023 Jul 14
Last detection date				
Wastewater	2024 May 21	2024 May 30	2024 May 20	2024 May 24
Clinical	2024 May 22	2024 May 7	2024 May 31	2024 Apr 23

*Values are no. (%) positive for each virus. NA, not applicable; RSV, respiratory syncytial virus. †Dates for combine surveillance were October 19, 2022–May 31, 2024; for the 2022–23 season, dates were October 19, 2022–June 30, 2023; and for the 2023–24 season, dates were July 1, 2023–May 31, 2024. ‡Eight SARS-CoV-2–positive clinical test results were detected in the 14 days before wastewater surveillance was initiated.

### SARS-CoV-2 Surveillance

**Figure 1:**
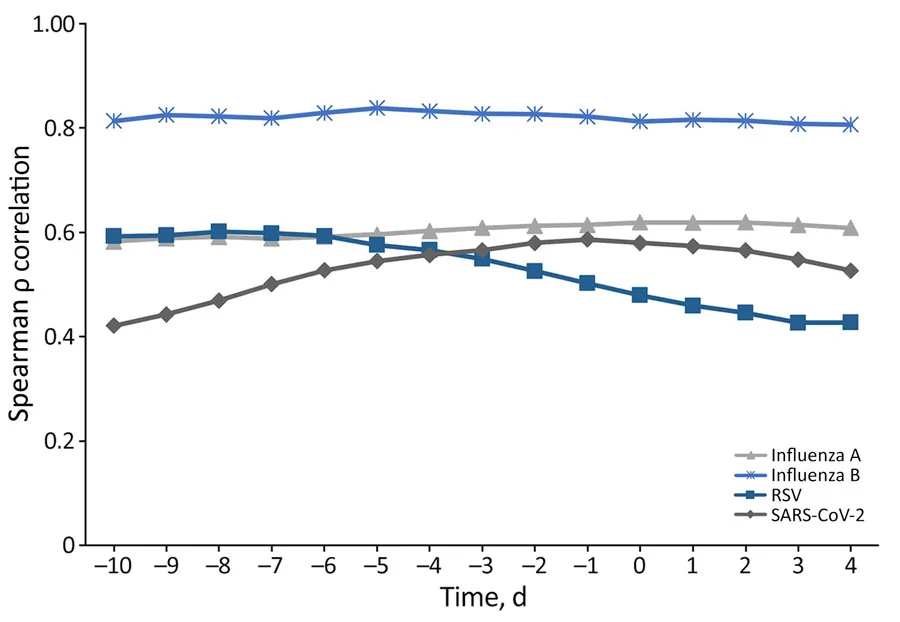
Time-lagged correlations between wastewater respiratory virus concentrations and clinical case counts in remote community, Alaska, USA, 2022–2024. Spearman rank-order correlation (ρ) assessed the association between positive clinical test counts and wastewater cycle threshold values for the number of days wastewater data shifted. For example, the value −7 on the x-axis indicates that wastewater values were shifted 7 days before (earlier) the clinical results from a given date. All correlation coefficients >0.17 are statistically significant at p<0.05. RSV, respiratory syncytial virus.

**Figure 2:**
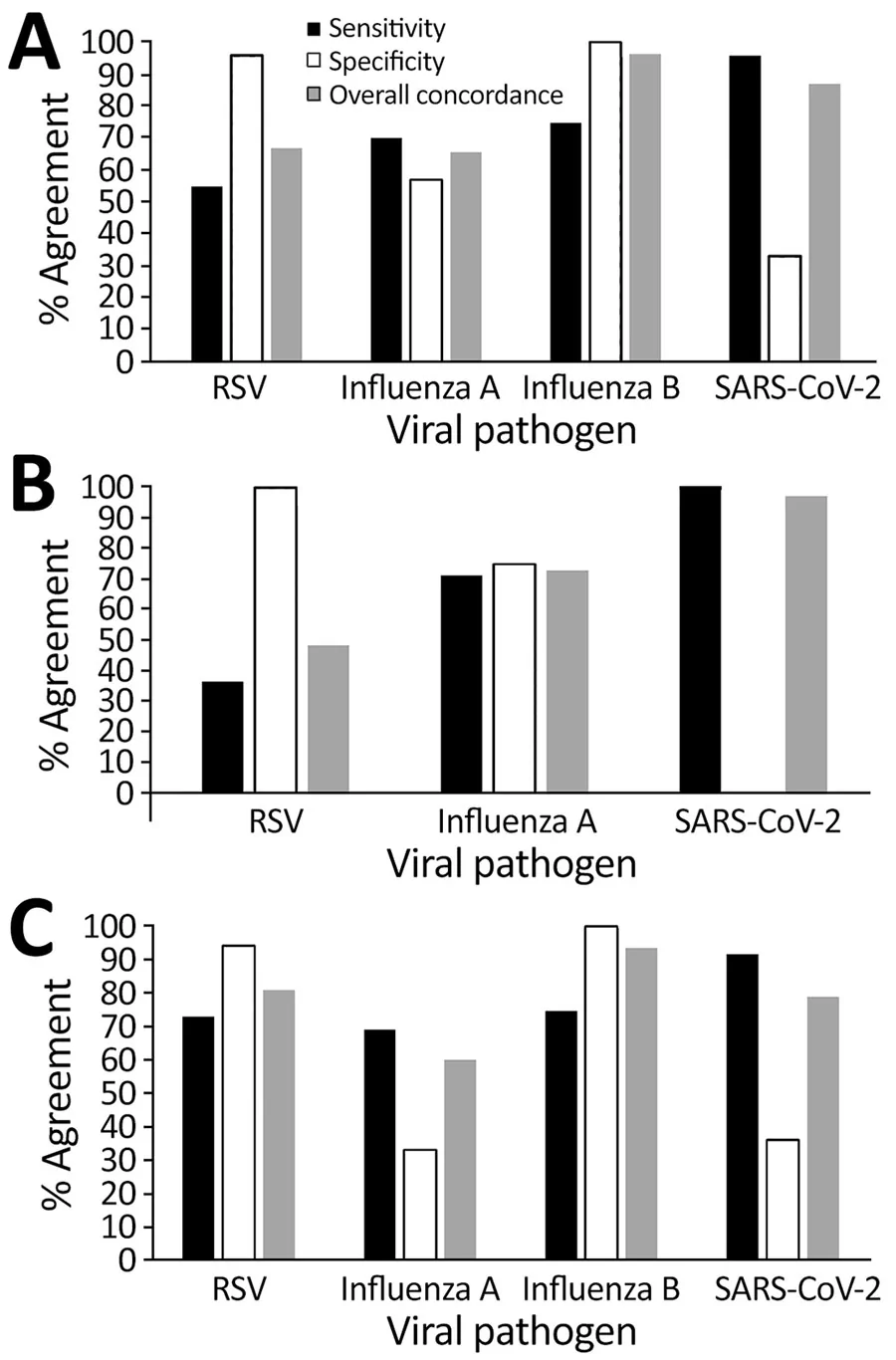
Sensitivity, specificity, and concordance of weekly wastewater testing results compared with weekly clinical testing from wastewater respiratory virus surveillance in remote community, Alaska, USA, 2022–2024. A) Overall study period 2022–2024; B) 2022–23 respiratory virus season; C) 2023–24 respiratory virus season. RSV, respiratory syncytial virus.

In wastewater, SARS-CoV-2 was detected on 91.4% (192/210) of sample days in the 2022–23 season and 73.0% (214/293) of sample days in the 2023–24 season (Table). For clinical samples, SARS-CoV-2 was detected 49.4% (126/255) of days in the 2022–23 season and 32.5% (106/326) of days in the 2023–24 season. In the 2022–23 season, clinical cases were detected throughout the year, and SARS-CoV-2 was detected year-round in wastewater. A notable peak in clinical case detections occurred in late January 2024, and a wastewater concentration peak occurred 8 days later. Correlation of SARS-CoV-2 wastewater and clinical test data was strongest with limited time shift (ρ = 0.59; wastewater shifted 1 day before clinical data) (Figure 1). An analysis using self-reported SARS-CoV-2 test results yielded a stronger correlation than observed with healthcare-based testing (Appendix Table 3). WS for SARS-CoV-2 was 95.9% sensitive and 87.1% concordant with weekly clinical testing results (Figure 2; Appendix Table 4).

### RSV Surveillance

**Figure 3:**
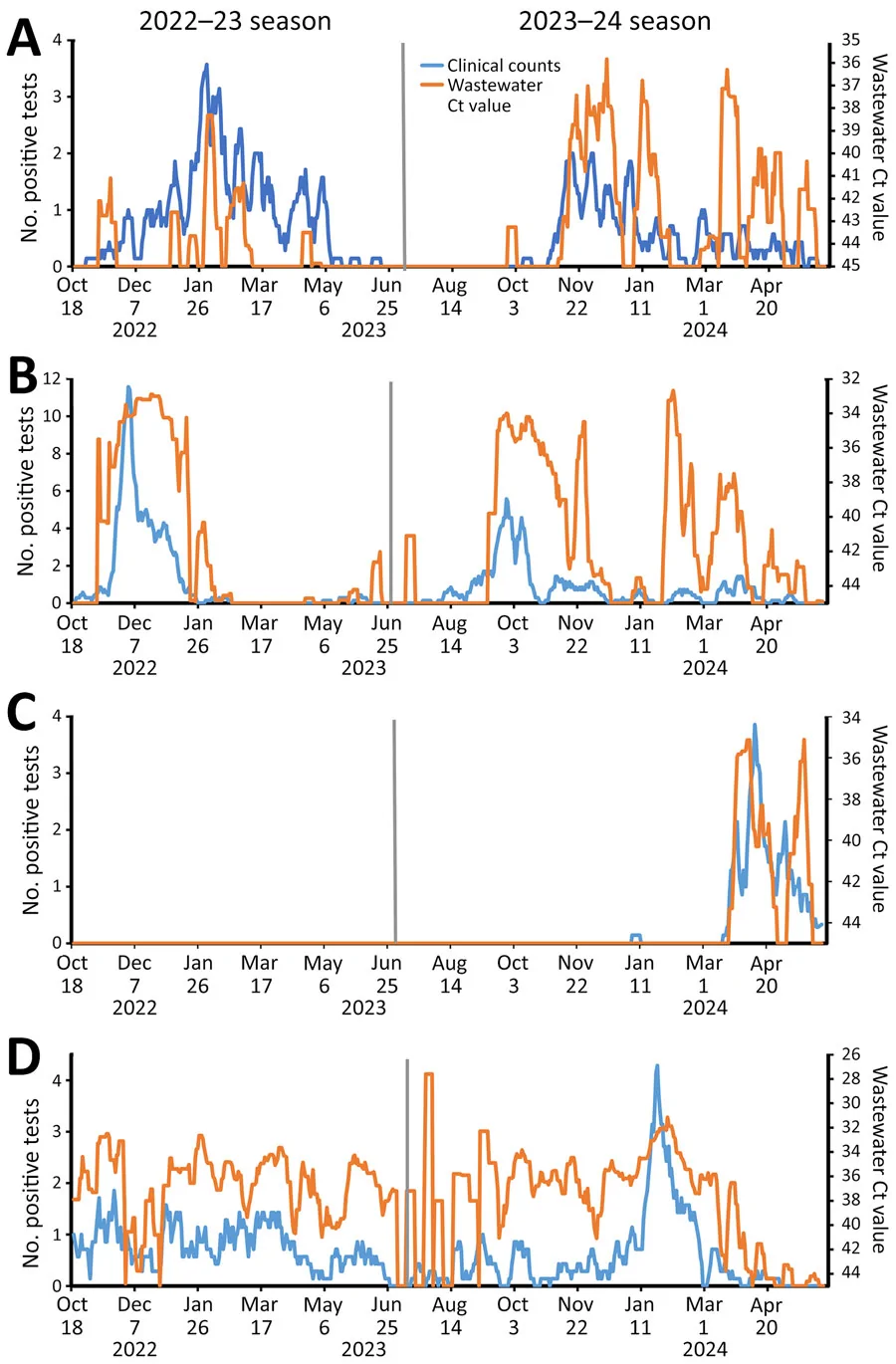
Daily 7-day moving average positive clinical tests and Ct values for respiratory virus surveillance in remote community, Alaska, USA, October 2022–May 2024. A) Respiratory syncytial virus; B) influenza A virus; C) influenza B virus; D) SARS-CoV-2. Ct, cycle threshold.

Overall, RSV was detected in wastewater on 22.1% (111/503) of surveillance days; observed wastewater positivity was lower in the 2022–23 season than in the 2023–24 season (13.8% vs. 28.0%) (Table). Laboratory-confirmed clinical RSV cases occurred on 37.0% (215/581) of days and observed daily clinical positivity was higher in the 2022–23 season than the 2023–24 season (46.7% vs. 29.4%). RSV circulation seasonality was similar across wastewater and clinical data. In the 2022–23 season, RSV clinical cases occurred during October 29, 2022–June 12, 2023, compared with RSV detection in wastewater during November 7, 2022–April 26, 2023 (Table; Figure 3). In the 2023–24 season, RSV was first detected in wastewater on September 26, 2023, and the first positive clinical test was on October 9, 2023. RSV was detected in wastewater through May 21, 2024, and by clinical testing through May 22, 2024. The correlation between the wastewater RSV signal and clinical testing data increased with increasing time shift of the wastewater data before the clinical data. The peak RSV correlation of 0.60 occurred with 8-day wastewater lead time. RSV correlations were stronger in the 2023–24 season than the 2022–23 season. WS for RSV was 55.0% sensitive and 67.1% concordant with weekly clinical testing results (Figure 2; Appendix Table 4). Sensitivity and concordance of RSV WS was markedly higher in the 2023–24 season: sensitivity increased from 36.7% to 73.3% and concordance increased from 48.6% to 81.3%.

### Influenza A Virus Surveillance

Wastewater testing detected influenza A virus on 36.8% (185/503) of surveillance days; detections were more frequent during the 2023–24 season (44.4%; 130/293 days) than the 2022–23 season (26.2%; 55/210 days). Influenza A virus–positive clinical tests occurred on 34.9% (203/581) of days (Table). Influenza A virus was first detected clinically on October 21, 2022, and in wastewater on November 7, 2022. Wastewater detection patterns mirrored clinical detection patterns (Figure 3). Both surveillance systems identified a wave of influenza A virus during the winter of 2022–23, followed by sporadic cases during the summer of 2023 and several subsequent waves of infection later in the 2023–24 season. We observed consistent, strong influenza A virus wastewater and clinical data correlations, and most lagged correlations were near or above 0.60 (Figure 1). Influenza A virus correlations were stronger during the 2022–23 season and peaked at 0.82 when the wastewater signal shifted 10 days before clinical signal (Appendix Table 3). WS for influenza A virus was 70.2% sensitive and 65.9% concordant with weekly clinical testing results (Figure 2; Appendix Table 4).

### Influenza B Virus Surveillance

Influenza B virus was not detected in wastewater or through clinical testing during the 2022–23 season. In the 2023–24 season, influenza B virus was detected in wastewater on 14.0% (41/293) of sample days and clinical tests were positive on 15.0% (49/326) of surveillance days. An initial clinical case of influenza B infection occurred in January 2024 with no concordant detection in wastewater. Additional clinical cases of influenza B infection were identified starting March 17, 2024, and in wastewater on March 21, 2024, and ongoing detection occurred through May 2024 (Figure 3). Influenza B virus wastewater and clinical data had correlations >0.80 across all time shifts and peaked at 0.85 when the wastewater signal shifted 5-days before clinical signal (Figure 1). WS for influenza B virus was 75.0% sensitive and 96.5% concordant with weekly clinical testing results (Figure 2; Appendix Table 4).

## Discussion

Implementation of WS by YKHC was unique in several respects. Bethel is a remote community off the road system with limited laboratory capacity and a sanitation system uncommon in the United States. At the time YKHC initiated WS, Alaska as a state lacked a coordinated WS program, necessitating sample shipment to a contracted laboratory, leading to additional costs, delays, and potential sample degradation. YKHC, as the regional Tribal healthcare and public health organization, was well positioned to locally implement WS. The YKHC Office of Environmental Health & Engineering had experience with water quality testing, were trusted by the community, had access to clinical testing data to validate WS, and were able to act on the information generated by WS. The simplicity of the cartridge-based rapid PCR testing system and its performance with unprocessed wastewater contributed to timely results; in most instances, samples were tested the same day they were collected. Local ownership of WS and the associated data likely mitigated community concerns and enhanced program sustainability (*16*).

Given uncertainties in the performance of WS in a subarctic, low water use, partially sewered community, YKHC used the 2022–23 season of WS to internally validate its performance. The strong correlations observed between clinical testing and wastewater results for influenza A virus and RSV during the 2022–23 season encouraged additional investment in WS and led to the creation of a public facing dashboard on the YKHC website (*17*). The dashboard provides background on WS, interpretation of the data, and suggests specific public health actions for community members depending on the wastewater results.

WS data showed moderate to high concordance with laboratory-confirmed clinical instances of respiratory viral infection. The strengths of the correlations were similar to those reported by a shorter study of COVID-19 WS in small, rural communities (*18*). Time-lagged correlation analysis suggests that wastewater detection of RSV could serve as a leading indicator of virus circulation in Bethel. Early identification of RSV circulation enables timely prevention through nirsevimab distribution and clinical and community information sharing. As a current best practice (*19*), YKHC does not use wastewater data in isolation but integrates it with other data sources, such as clinical data, for public health decision making.

Several factors could explain observed discordances between wastewater and clinical data. Wastewater detections in the absence of positive clinical tests suggest circulating virus in the community but no persons ill enough to seek healthcare and receive clinical testing. In addition, some persons shed viral material for prolonged periods following infection, as noted for SARS-CoV-2 (*20*), resulting in wastewater detection in the absence of new clinical cases. A positive clinical test in the absence of a detectable wastewater signal might be because waste from the ill person has not entered the centralized sewer system, viral RNA was diluted in wastewater to below the limit of detection for the assay, or PCR inhibitors were in the wastewater (*21*).

A strength of this study is evaluation of 2 years of comprehensive clinical testing and wastewater data, resulting in a robust dataset. The first limitation of our study is that identification of clinical infections relied upon community members seeking healthcare and receiving viral testing, likely leading to underascertainment of infections. However, YKHC provided care for almost everyone in the region operating the only hospital and emergency department in the Yukon-Kuskokwim Delta. YKHC standard of care was to test all persons with respiratory symptoms, which provided a comprehensive clinical picture of SARS-CoV-2, influenza, and RSV circulation in the community. A second potential limitation common to WS evaluations was the possible mismatch between the population accessing clinical testing and the population contributing to the sampled wastewater. Bethel serves as the medical and transportation hub for the region, potentially leading to mismatches in persons receiving clinical testing and contributing to the wastewater system. However, the remote nature of Bethel, which is off the road system and primarily accessed by plane, likely minimizes that population mismatch compared with road-connected communities in the United States with a variety of clinical testing options. Our analysis only used clinical test results from the YK Delta Regional Hospital, meaning all clinical test results came from persons physically in Bethel who could contribute to the city’s wastewater. A third limitation relates to the unique sanitation system in Bethel, where sewage stored at the household level might be introduced into the centralized sewage system days to weeks after it was created. That delay could weaken correlations with clinical testing or decrease the sensitivity of WS because of decay of the viral biomarkers in the wastewater. To explore that issue, we conducted lagged correlational analyses, which found the strongest correlations when the wastewater results were shifted earlier in relation to clinical testing. A fourth limitation is that the passive wastewater sampling method yielded semiquantitative viral wastewater concentrations. Thus, we report Ct values and not estimated viral concentrations.

In summary, YKHC implemented WS in a rural and remote community midway through the COVID-19 pandemic. That surveillance system frequently identified respiratory viruses in wastewater and demonstrated statistically significant correlations with clinical testing. WS provided YKHC respiratory virus surveillance information that complemented and corroborated traditional clinical surveillance data. WS data helped inform YKHC public health decision-making related to the timing of influenza vaccine campaign and the distribution of nirsevimab, a long-lasting monoclonal antibody used to protect infants from RSV, and supported education and messaging for patients and clinicians. New components of the YKHC WS program include testing for additional respiratory and enteric pathogens, exploratory efforts to enhance active case finding of tuberculosis, and expansion of WS to remote villages in the region. YKHC demonstrated the feasibility of WS in rural Alaska and that correlation between WS and clinical results can support and inform public health activities to protect the community.

Appendix Additional information on wastewater respiratory virus surveillance in remote community, Alaska, 2022–2024.
