# Supplementary material for: Wastewater Respiratory Virus Surveillance in Remote Community, Alaska, USA, 2022–2024
Source: Emerg Infect Dis. 2026 Aug;32(Suppl 1):S71–9. doi: 10.3201/eid3213.260709 (PMC13585681; doi:10.3201/eid3213.260709)
Supplement: Supplementary file 1 [file 26-0709-Techapp-s1.pdf]

Article DOI: https://doi.org/10.3201/eid3213.260709

EID cannot ensure accessibility for supplementary materials supplied by authors. Readers who have difficulty accessing supplementary content should contact the authors for assistance.

Wastewater Respiratory Virus Surveillance in Remote Community, Alaska, USA, 2022–2024

Appendix

Wastewater sample collection

Moore swabs were made from individually packaged sterile 4-inch by 4.1-yard cotton gauze rolls, which were unrolled, loosely bunched, and tied in the middle. The swab was attached with fishing line to a weighted chain hung in the wastewater stream passing through the lift station. This allowed the swab to float freely in the wastewater stream and absorb particulate matter while staying clear of the lift station pumps.

For safety, two people set the swab. One person pulled the chain to the surface while the other secured the swab's fishing line to the chain ≈12 inches above the weight. The swab was then lowered into the wastewater, and the hatch was closed. Retrieval involved cutting the fishing line and placing the swab in a new resealable plastic bag. Wastewater was squeezed from the swab into a pre-labeled sterile sample bottle through a cut corner of the bag. The sample was placed in a transport cooler and immediately taken to the lab for processing.

Nucleic acid concentration in wastewater samples

We adapted the concentration process (Method B) described by Daigle et al. (*1*) Briefly, we mixed 40 uL of Tween 80 solution with 40 mL of untreated wastewater in a vortex mixer for 20 seconds on the highest speed. Samples were clarified in a centrifuge for 32 minutes at 2900 RFC (relative centrifugal force). We then decanted 15 mL of the supernatant to an Amicon Ultra15 10-kDa centrifugal filter tube (MilliporeSigma, Burlington, MA). The filter tube was centrifuged for 51 minutes at 2900 RFC. After the second centrifuge cycle, we transferred the

concentrated sample to a clean 2 mL tube and added 150 uL of PCR-grade water. We used 300 uL of the concentrated wastewater sample for analysis in Cepheid GeneXpert cartridge following the manufacturer's protocol for testing clinical specimens.

We compared virus-specific PCR results for wastewater samples tested with and without concentration (N = 119 paired samples). The proportions of wastewater samples with detectable SARS-CoV-2, influenza A, and RSV did not vary by preparation method (Appendix Table 1). The mean PCR Ct value for influenza A was 0.59 cycles lower in the unconcentrated samples (Appendix Table 2). For SARS-CoV-2 the concentrated samples demonstrated a mean Ct 0.56 cycles lower than the unconcentrated samples. We observed no statistically significant difference in mean Ct values for RSV by sample preparation method.

References

1. Daigle J, Racher K, Hazenberg J, Yeoman A, Hannah H, Duong D, et al. A sensitive and rapid wastewater test for SARS-COV-2 and its use for the early detection of a cluster of cases in a remote community. Appl Environ Microbiol. 2022;88:e0174021. PubMed https://doi.org/10.1128/aem.01740-21

Appendix Table 1. Proportions of wastewater samples with respiratory virus detection by preparation method and virus

Virus	Matched dates (N)	Both detected (n)	Concentrated only (n)	Unconcentrated only (n)	Both non-detect (n)	p value†
Influenza A	119	27	6	4	82	0.75
RSV	119	5	9	14	91	0.40
SARS-CoV-2	119	110	6	3	0	0.51

*Influenza B was not detected by either preparation method on the 119 dates.
†Exact McNemar test.

Appendix Table 2. Respiratory virus polymerase chain reaction (PCR) cycle threshold (Ct) wastewater values by sample preparation method and virus

		Unconcentrated		Concentrated		Comparison	
Virus	No. matched samples	Mean Ct (SD)	No. detected (%)	Mean Ct (SD)	No. detected (%)	Ct mean difference (95% CI)	p value†
Influenza A	119	42.96 (4.03)	31 (26.1)	43.55 (2.95)	33 (27.7)	−0.59 (−0.98, −0.19)	0.0038
RSV	119	44.17 (2.41)	19 (16.0)	44.54 (1.46)	14 (11.8)	−0.37 (−0.82, 0.08)	0.11
SARS-CoV-2	119	36.62 (3.54)	113 (95.0)	36.05 (2.90)	116 (97.5)	0.56 (0.07, 1.06)	0.025

*Influenza B was not detected by either preparation method on the 119 dates. Ct, cycle threshold.
†Paired *t*-test.

Appendix Table 3. Peak lagged correlations between wastewater respiratory virus concentrations and clinical case counts by season for four respiratory viruses in Bethel, Alaska, 2022–2024*

		Correlation coefficient (days lag between wastewater and clinical cases)†		
Virus	Case definition	Total Study Period (2022 Oct 19–2024 May 31)	Season 2022–23 (2022 Oct 19–2023 Jun 30)	Season 2023–24 (2023 Jul 1–2024 May 31)
RSV	Positive clinical test	0.60 (8 d before)	0.47 (1 d after)	0.69 (7 d before)
Influenza A	Positive clinical test	0.62 (1 d after)	0.82 (10 d before)	0.48 (4 d before)
Influenza B	Positive clinical test	0.85 (5 d before)	NA	0.85 (5 d before)
SARS-CoV-2	Positive clinical test	0.59 (1 d before)	0.56 (1 d before)	0.66 (2 d after)
	Self-reported test	0.65 (3 d before)	0.58 (4 d before)	0.73 (2 d before)

* NA. not applicable; RSV, respiratory syncytial virus.

†Correlations between wastewater virus concentration and clinical detection presented as absolute values of the Spearman correlation coefficient; the raw calculated coefficient is negative because the wastewater Ct value is inversely related to viral concentration. Lag time is the number of days the wastewater data was shifted relative to the clinical data. For example, “8 days before” signifies the peak correlation occurred when the wastewater data was shifted 8 d earlier relative to the clinical testing data.

Appendix Table 4. Diagnostic performance of wastewater testing for RSV, SARS-CoV-2, influenza A, and influenza B in comparison to clinical test results in Bethel, Alaska, October 2022–May 2024*

		Wastewater and clinical testing contingency results (weeks)†						
Virus	Season	WW+ Clinical+	WW– Clinical+	WW– Clinical–	WW+ Clinical–	% Concordance (95% CI)‡	% Sensitivity (95% CI)	% Specificity (95% CI)
RSV	Overall	33	27	24	1	67.1 (56.5–76.1)	55.0 (42.5–66.9)	96.0 (80.5–99.3)
	Season 2022–23	11	19	7	0	48.6 (33.4–64.1)	36.7 (21.9–54.5)	100 (64.6–100.0)
	Season 2023–24	22	8	17	1	81.3 (68.1–89.8)	73.3 (55.6–85.9)	94.4 (74.2–99.0)
Flu A	Overall	40	17	16	12	65.9 (55.3–75.1)	70.2 (57.3–80.5)	57.1 (39.1–73.5)
	Season 2022–23	15	6	12	4	73.0 (57.0–84.6)	71.4 (50.0–86.2)	75.0 (50.5–89.8)
	Season 23/34	25	11	4	8	60.4 (46.3–73.0)	69.4 (53.1–82.0)	33.3 (13.8–60.9)
Flu B	Overall	9	3	73	0	96.5 (90.1–98.8)	75.0 (46.8–91.1)	100 (95.0–100.0)
	Season 2022–23	0	0	37	0	100.0 (90.6–100.0)	NA	100 (90.6–100.0)
	Season 2023–24	9	3	36	0	93.8 (81.8–98.0)	75.0 (46.8–91.1)	100 (90.4–100.0)
SARS-CoV-2	Overall	70	3	4	8	87.1 (78.3–92.6)	95.9 (88.6–98.6)	33.3 (13.8–60.9)
	Season 2022–23	36	0	0	1	97.3 (86.2–99.5)	100 (90.4–100.0)	0 (0.0–79.3)
	Season 2023–24	34	3	4	7	79.2 (65.7–88.3)	91.9 (78.7–97.2)	36.4 (15.2–64.6)

*NA, not applicable; RSV, respiratory syncytial virus; WW, wastewater.

†Contingency testing results by virus for a surveillance week, e.g., WW(-) Clinical(+) indicates the number of weeks that the wastewater samples tested negative when there were positive clinical tests for a given virus. CI, confidence interval; Flu A, influenza A; Flu B, influenza B; RSV, respiratory syncytial virus; WW, wastewater; + indicates a positive test result; - indicates negative test result.

‡Clinical test results were the referent. Concordance = (true positive weeks + true negative weeks)/total surveillance weeks; sensitivity = true positive weeks/(true positive weeks + false negative weeks); specificity = true negative weeks/(true negative weeks + false positive weeks)

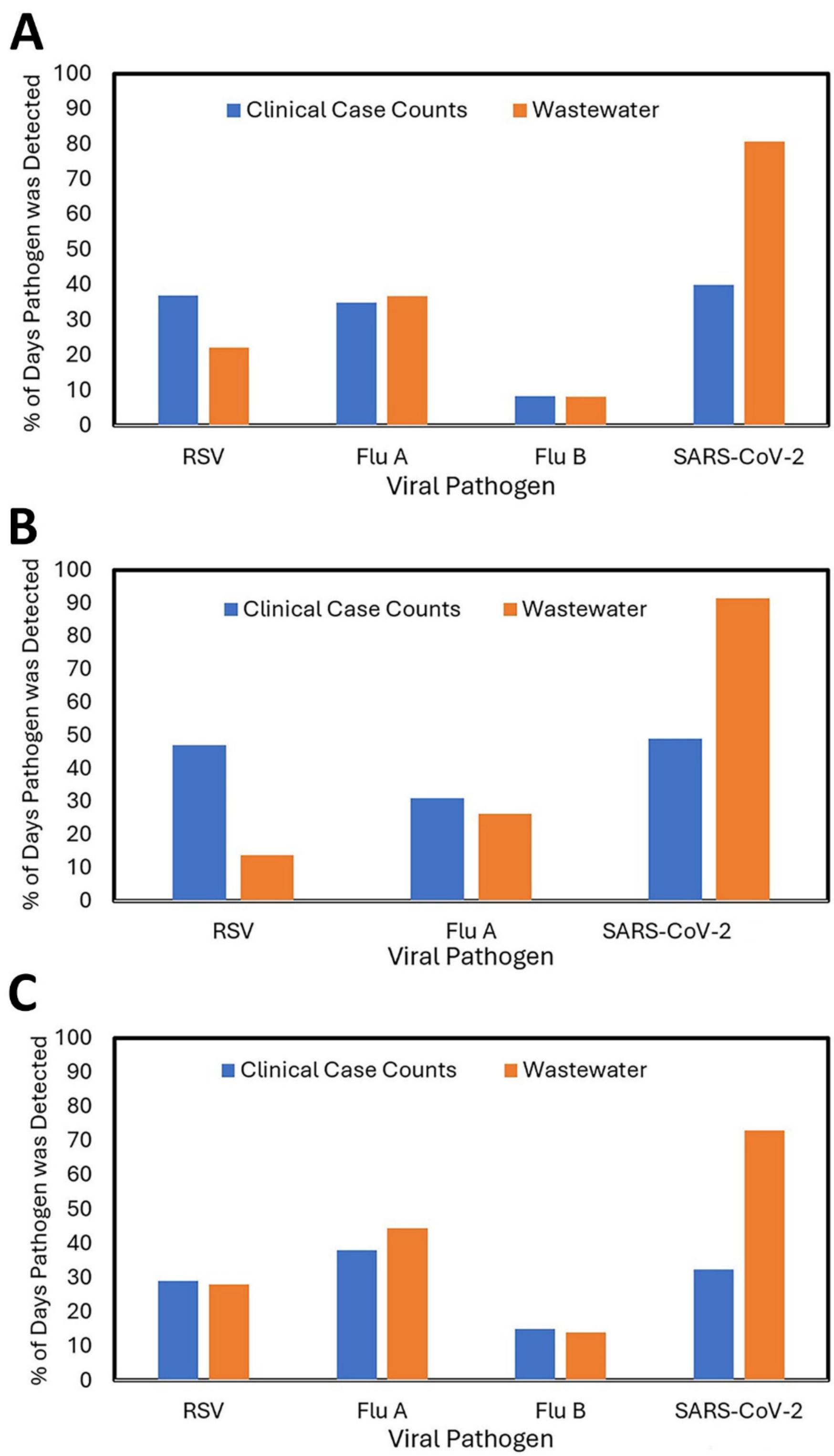

Appendix Figure. The proportion of sample days that each viral pathogen was detected by wastewater surveillance (orange bar) and in routine clinical testing (blue bar) by season: A) Overall 2022–2024; B) Season 2022–2023; and C) Season 2023–2024.
